# An ecologically friendly process for graphene exfoliation based on the “hydrodynamic cavitation on a chip” concept†

**DOI:** 10.1039/d1ra03352b

**Published:** 2021-05-18

**Authors:** Mohammad Jafarpour, Araz Sheibani Aghdam, Moein Talebian Gevari, Ali Koşar, Mustafa Kemal Bayazıt, Morteza Ghorbani

**Affiliations:** Sabanci University Nanotechnology Research and Application Center 34956 Tuzla Istanbul Turkey; Faculty of Engineering and Natural Science, Sabanci University 34956 Tuzla Istanbul Turkey; Division of Solid State Electronics, Department of Electrical Engineering, The Ångström Laboratory, Uppsala University 75237 Uppsala Sweden; Center of Excellence for Functional Surfaces and Interfaces for Nano-Diagnostics (EFSUN), Sabanci University Orhanli, 34956, Tuzla Istanbul Turkey; Department of Biomedical Engineering and Health Systems, KTH Royal Institute of Technology SE-141 57 Stockholm Sweden mortezag@kth.se

## Abstract

Tremendous research efforts have recently focused on the synthesis of graphene from graphitic materials, while environmental issues, scalability, and cost are some of the major challenges to be surmounted. Liquid phase exfoliation (LPE) of graphene is one of the principal methods for this synthesis. Nevertheless, sufficient information about the mechanisms of exfoliation has yet to emerge. Here, a microreactor based on the hydrodynamic cavitation (HC) on a chip concept is introduced to exfoliate graphite in a totally green process which involves only natural graphite flakes and water. HC-treated graphitic materials were characterized by UV-Vis and Raman spectroscopy, DLS (Dynamic Light Scattering), AFM (Atomic Force Microscopy), and SEM (Scanning Electron Microscopy) analyses. The present sustainable reactor system was found to exfoliate thick and large graphite particles to nano-sized sheets (∼1.2 nm) with a lateral size of ∼500 nm to 5 μm.

## Abstract 2

LPE of graphene with a hydrodynamic cavitation microreactor is a totally green process which involves only natural graphite flakes and water.

## Graphical abstract

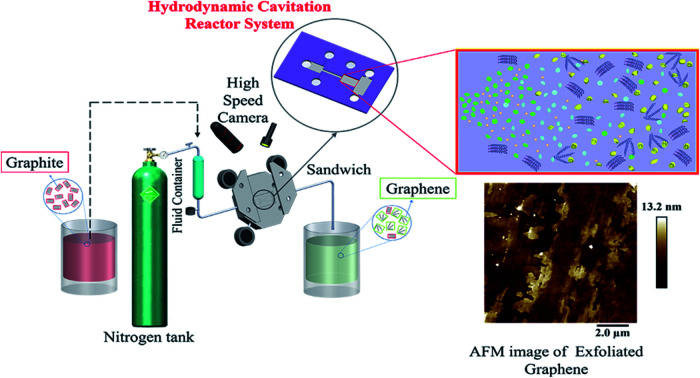

## 1. Introduction

2D nanomaterials have been extensively implemented for improvements in the quality of life since their discovery and development. During the past decade, many research efforts have been focused on graphene due to its outstanding electrical, optical, chemical, and mechanical properties, as well as its unique 2D honeycomb lattice.^1,2^ These unique characteristics have attracted the attention of many researchers so that the scope of the research on graphene has been broadened beyond materials engineering and physics.^3^ In this regard, a substantial number of studies on drug delivery,^4,5^ nanoelectronics,^6,7^ batteries and fuel cells,^8,9^ sensors,^10,11^ and supercapacitors^12^ have been recently published.

Production of graphene and related materials at a reasonable cost from graphite is a long-lasting scientific challenge and puts an obstacle against graphene-based emerging applications. The properties of graphene and related materials are closely related to their synthesis method, which could enable the precise control of their shape, size, and surface properties, thereby leading to versatile physical, chemical, and biomedical characteristics.^13^ Over the years, various methods have been proposed to produce graphene, which can be categorized into two major approaches: top-down and bottom-up.^14^ Top-down approaches involve the separation of the stacked layers of graphite into graphene sheets. Mechanical exfoliation,^15,16^ liquid-phase exfoliation (LPE),^17,18^ unzipping of multi-wall carbon nanotubes (MWCNTs),^19,20^ and arc discharge^21,22^ are some examples of this approach. In contrast to top-down methods, bottom-up methods involve synthesizing graphene from carbon-containing sources such as chemical vapor deposition,^23,24^ epitaxial growth,^25^ and pyrolysis.^26^

In general, in top-down methods, the process involves mechanical and chemical energies to break down weak van der Waal forces in high-purity graphite sheets.^27,28^ Typically, the mechanical routes for exfoliation of flakes apply in the form of two forces. The vertical impacts act on the flakes, which causes to overcome energy between layers to peel them apart (normal force), and the sliding relative movements between layers occur due to the exerted lateral force (shear force).

LPE, one of the most widely used top-down methods for graphene production, was first introduced in 2008. In this method high energy sonication or mixing are conducted to exfoliate graphite to graphene sheets in a solvent with addition of surfactants to avoid oxidation or reduction. On the other hand, spontaneously exfoliation of graphene sheets under the effect of high energy liquid–liquid interface is another approach, where graphite particles act as a surfactant in aqueous based medium.^29,30^ Generally, there are three main steps in LPE synthesis: (i) dispersion of graphite in a suitable solvent, (ii) exfoliation, and (iii) purification of the final products.^31^ In the second step, the formation and collapse of bubbles on the flake surfaces instantly result in a compressive stress wave propagation throughout the particle. Based on the theory of stress waves, the particle is also exposed to a reflected tensile stress wave. The cycle of creation and collisions of bubbles leads to intensive tensile stress in the flakes. Additionally, the other potential scenario is the exertion of unbalanced lateral compressive stress. This kind of stress can also break down adjacent layers by the shear effect.^32^ As a result, it is an efficient and fast approach to develop nano-sized particles, where the prominent role belongs to cavitation bubbles.^33^

Cavitation is a phase change phenomenon involving the nucleation, growth, and collapse of gas or vapor-filled bubbles in liquids.^34,35^ The collapsing bubbles (cavities) in the liquid provide the energy source to initiate and enhance a wide range of chemical processes and introduce physical effects to break down graphite layers into graphene.^36^ The resulting bubble collapse could generate very high energy densities (energy per unit volume), which causes a rise in the local temperature and pressure as large as 5000 K and 500 atm, respectively, over an extremely short period of time.^37^ In general, acoustic-based exfoliation is carried out with an ultrasonic water bath or probe-tip sonicator, which can be scaled up to no more than a few hundred milliliters.^38^ Indeed, because of the inefficient energy transfer from the source to the liquid medium, the increase in the volume will exacerbate the production rate. Thus, exfoliation of graphite to graphene by ultrasonication is not a suitable way for large scale graphene production.^39^

Due to the significance of hydrodynamic cavitation in fluidic systems, many studies have been dedicated to provide an understanding about the effects of major parameters such as thermophysical properties of the working fluid, geometry of the reactor, and surface roughness elements.^40,41^ Recently, the generation of hydrodynamic cavitating flows in microfluidic devices has gained much attention because of the scalability, cost-effectiveness, and energy-efficiency. Furthermore, facile flow generation processes besides the stationary section of hydrodynamic reactors make them even more popular and effective.^42,43^

Some studies on liquid exfoliation inside a microreactor are capable of generating hydrodynamic cavitation. For example, Liu *et al.*^44^ attempted to prepare single and few-layered graphene flakes in a cavitation reactor by employing a water–acetone mixture. Their process yield was 4%, and they introduced this method as a promising mass production tool with advantages of low cost and green process. In one of the recent studies conducted by Qiu *et al.*,^45^ a 50 g L^−1^ graphite suspension with a sufficient amount of surfactant (sodium cholate) was processed by passing around 2000 times through a microreactor (∼3 hours). The hydraulic power and relative energy consumption of their system were about 5 W and 2 MJ L^−1^, respectively. They reported that the surfactant might undergo destruction under intense cavitation, which can prevent the increase in the yield of process. In another study, graphene and its analogues materials were produced by the use of liquid phase exfoliation and microreactor, where Yi *et al.*^46^ introduced the fluid dynamics method for scalable and efficient production. They performed the experiments with the help of a high-pressure plunger pump, and the suspensions were under the effect of *N*,*N*-dimethylformamide (DMF) as a dispersion medium. They treated the working fluid in 5-cycles.

Motivated by the emerging studies on LPE in microfluidic devices, herein, a sustainable hydrodynamic cavitation reactor system with a nozzle, which lead to a sudden decrease in the cross-sectional area of the fluid path and an increase in the velocity of the working fluid, was designed. This system was shown to be highly efficient in the large-scale preparation of stable graphene solutions from natural graphite powder in water. Accordingly, we developed an eco-friendly hydrodynamic cavitation induced microreactor, which could exfoliate graphene with the use of just pure water instead of harmful and expensive solvents and chemicals.

## 2. Materials and methods

### 2.1. Chemicals and materials

Natural graphite powder was purchased from Alfa Aesar (graphite flake, natural, −10 mesh, LOT: U24E068). The graphite solution with 25 mg L^−1^ solid concentration was prepared using de-ionized water without the use of any surfactant or dispersant agent. In a typical experiment, graphite flakes in water were sonicated using an ultrasonic bath sonicator (Bandelin Sonorex, Rangendingen, Germany) for 30 min. The resulting graphite solution was kept on a side for 15 min to precipitate out the unstable large graphite flakes, and the supernatant (so-called as ‘the starting graphite dispersion’) was separated to be used in the hydrodynamic cavitation reactor, where it was passed through the reactor.

### 2.2. Microfluidic device geometry and fabrication

The microfluidic device (hydrodynamic cavitation reactor) used in this study was fabricated using the semiconductor microfabrication techniques on silicon and was bonded to a glass cover to make sure that the reactors are leakproof. Thus, a fixed upstream pressure can lead to a stable flowrate in the reactors. The fabricated reactor consists of three main regions, namely inlet, nozzle, and extension zone. The widths of the inlet and extension are identical, while the width of the nozzle is smaller so that a sudden decrease in the flow cross-sectional area can be achieved.

According to the Bernoulli's principle, velocity and static pressure are inversely related. Hence, the increase in the fluid velocity as a result of the change in geometry of the flow restrictive element in the reactor leads to a decrease in the static pressure, which triggers the formation of cavitating flows.

Since the energy released from the collapsing bubbles provides the input of our system, it is vital to make sure that the majority of the bubble collapse occurs inside our reactor. For this purpose, the nozzle length in our reactor is significantly longer than the available studies in the literature, which facilitates the pressure recovery within the system. Hence, the bubbles face a relatively high-pressure region in the extension area of the reactor, which results in the collapse within the reactor. The second feature of the fabricated reactor lies on the engineered wall of the nozzle area, where roughness elements were formed. Our previous studies reported that the presence of roughness elements on the walls facilitated the formation of cavitating flows.^47^ The microfluidic device in this study is also equipped with wall roughness elements so that hydrodynamic cavitation can incept at lower upstream pressures. The reactor is resistant to high pressures and can withstand very high upstream pressures up to 1200 psi.

As mentioned before, the microfluidic device in this study consists of three regions with the same length of 2000 μm. The widths of inlet and extension zones are 900 μm, while this value for the nozzle is 400 μm. 2/3*L*_n_ of the nozzle length is equipped with triangular roughness elements with a height of 4 μm. One inlet and two outlets are formed on the reactor to realize the flow path. The detailed geometrical parameters of the microfluidic device are listed in Table 1.

**Table 1:** The geometrical characteristics of the reactor

Physical configuration	Range
Nozzle length (*L*_n_)	2000 μm
Nozzle width (*W*_n_)	400 μm
Hydraulic diameter (*D*_h_)	233 μm
Extension region length	2000 μm
Extension region width	900 μm
Length of the roughness elements (*L*_R_)	2/3*L*_n_
Height of the roughness elements (*H*_R_)	0.01*W*_n_

The process flow of the fabrication of the reactor in this study is the repetition of material deposition, patterning, and material removal using the standard microfabrication techniques. Accordingly, a layer of silicon dioxide was deposited on a double-side-polished silicon wafer. The inlet and outlet ports were patterned on the surface by the photolithography and dry etching processes. Then, second photolithography and deep reactive ion etching (DRIE) were performed on the wafer to obtain the final design. The silicon wafer was then bonded anodically to a glass cap to finalize the nozzle configurations. Three pressure ports were patterned on the surface along with the inlet and outlet ports to assist in the measurements of the static pressure at the inlet, nozzles, and extension zones. More detailed information about the fabrication process flow can be found in our previous studies.^48^

### 2.3. Preparation of graphene nanosheets

**Fig. 1:**
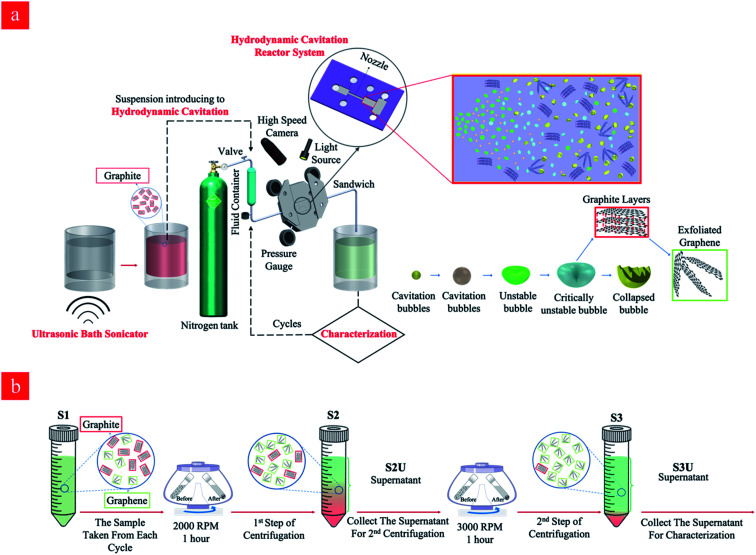
(a) The schematic of the hydrodynamic cavitation reactor system for the production of graphene nanosheets, and (b) the sequential centrifugation method for the isolation of the stable graphene nanosheets produced in the hydrodynamic cavitation reactor system.

Graphene nanosheets were prepared using a hydrodynamic cavitation reactor system (shown in Fig. 1a), which was constructed in similar lines with our previous studies.^47^ The working fluid (the starting graphite dispersion) was kept in a stainless-steel container (1 gallon), which was connected to a high-pressure pure nitrogen tank, was introduced to the system *via* proper fittings and stainless-steel tubing. The microfluidic device was installed and sandwiched into a homemade aluminum package, which facilitated flow visualization and prevented any leakage. The sandwich holder consists of one inlet connected to the fluid container and one outlet, where the fluid leaves the reactor. The pressure sensors (Omega, Manchester, UK) were also installed on the package to measure the static pressures at three different locations of the reactor. A double-shutter CMOS high-speed camera (Phantom v310) along with a macro camera lens with a focal length of 50 mm was used to record the flow patterns during the experiments, while the volumetric flowrate of the system was measured at different upstream pressures.

The prepared solution was introduced to the tubing system by applying the upstream pressure supplied by the nitrogen tank. The solution was propelled to the hydrodynamic cavitation reactor, where the exfoliation process happened in the nozzle and extension regions. The increase in the upstream pressure leads to a faster fluid flow in the system. One of the major parameters, Reynolds number, is expressed as:1
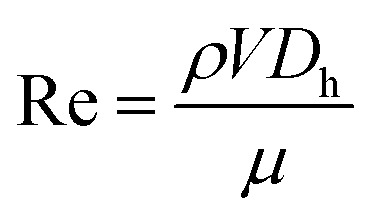
where *ρ* and *μ* are the fluid density and dynamic viscosity, respectively. The density of water at 20 °C is 998.2 kg m^−3^, and the dynamic viscosity is 1 cP in this study. Since the concentration of the graphite suspension is low, its effect on the density and viscosity of this working fluid is neglected. The velocity of the system, on the other hand, is calculated from the measured volumetric flow rate and cross-sectional area. *D*_h_ is the hydraulic diameter of the nozzle. Cavitating flow characterization is of great importance to assess the intensity and flow pattern formation. For this purpose, the cavitation number is used and defined as:2
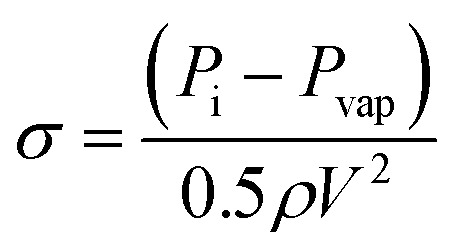
where *P*_i_ is the upstream pressure, *P*_vap_ is the saturation vapor pressure of the working fluid, *V* is the characteristic velocity of the fluid in the reactor, which is calculated at the beginning of the nozzle based on the volumetric flow rate of the system (flow rate/cross-sectional area).

### 2.4. Characterization methods

After different cycles of hydrodynamic cavitation, the collected samples were subjected to sequential centrifugations (Allegra X-15R, Beckman Coulter, Fullerton, CA, USA) to remove any unexfoliated material. The procedure for the sequential centrifugations is given in the Discussion section. The optical microscopy, Raman spectroscopy, scanning electron microscopy (SEM), and atomic force microscopy (AFM) for the samples were performed by transferring several drops of the supernatant (the top two-thirds of the dispersion from S3U samples) of second centrifuged suspension on silicon wafer substrates. The microscopic size and morphology of graphite/graphene were characterized by optical microscopy (Leica DM2700 M, Germany) and SEM (FE-SEM, LEO Supra VP-55, Germany). SEM images were taken after coating a very thin layer of gold–palladium alloy to observe the physical morphology and thickness of existing layers of the graphene. AFM measurements of graphene were made under ambient conditions at 60% relative humidity and 22 °C with a Digital Instruments Bruker Multimode 8 in tapping mode. The characterization was obtained using a NanoAndMore tip with a bending spring constant of 40 N m^−1^, resonance frequency of 50–200 kHz, and tip radius of 10–20 nm. UV-visible measurements were conducted on the samples in disposable cuvettes using a double-beam device (Varian Cary 5000 UV/Vis-NIR spectrometer) in the range of 200–800 nm. Raman spectroscopy was performed on a Renishaw inVia Reflex with the laser frequency of 532 nm as an excitation source. Raman spectra were obtained and normalized from at least 15 different spots on each sample. The size distribution of flakes after the specific cycles was determined using the dynamic light scattering (DLS) method. In this method, 1 mL of each sample was characterized in disposal cuvettes. The experiment was carried out with a Zetasizer Nano ZS (Malvern Instruments) device equipped with a He/Ne laser operating at 633 nm as a light source.

## 3. Results and discussion

Graphene nanosheets were produced in the hydrodynamic cavitation reactor system (Fig. 1a), where a top-down approach was adapted, and natural graphite flakes were exfoliated by energy released from the collapse of the cavitation bubbles. It is worth noting that the hydrodynamic cavitation-assisted production of graphene nanosheets in water is a green and sustainable process since it does not use any kind of chemicals such as surfactants and/or stabilizers. In this process, graphite particles act as a solid interface in the working fluid and facilitate the heterogeneous bubble nucleation so that the process had low input energy for cavitation generation. The method is a fast and energy-efficient production method, where the aqueous dispersions of graphite are treated through the cavitation setup, and the process lasts just a few seconds. The current hydrodynamic cavitation reactor system relying on a single nozzle microreactor is able to produce ∼3.125 mg of graphene in a day, however, the production may be scaled up from milligrams to kilograms by engineering parallel multichannel chips with multi-nozzle microreactors.

### 3.1. Hydrodynamic cavitation and flow patterns

Under cavitating flow conditions, the static pressure at the nozzle area drops to a critical value due to a sudden change in the flow geometry. The high-speed camera system captures cavitating flows at the beginning of the nozzle area. The upstream pressure (*P*_i_) corresponding to cavitation inception is 350 psi. The corresponding flow velocity is 68.2 m s^−1^, while the corresponding Reynolds number can be found as 15 861. Thus, it is evident that the flow is turbulent even at cavitation inception.

**Fig. 2:**
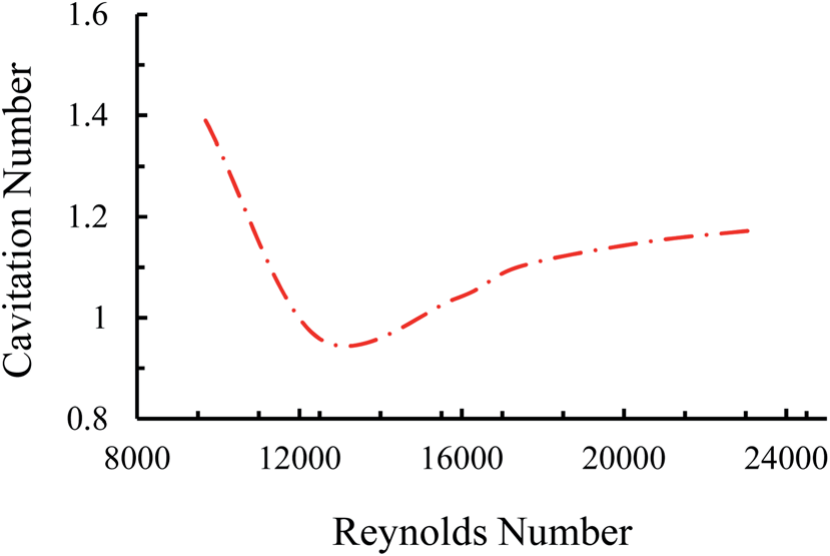
Cavitation number as a function of Reynolds number at different applied pressures.

Four main cavitating flow regimes could be observed under different conditions, namely, inception, developed flow, supercavitation, and choked flow. The inception of the cavitating flow appears when the gas phase is generated and corresponds to the weakest cavitating flow and largest cavitation number. With a gradual increase in the upstream pressure, the velocity increases so that the cavitation number follows a decreasing trend. At some points, when the reactor is saturated with the fluid flow, the velocity does not increase any more with the upstream pressure. Beyond this point, the cavitation number has an increasing trend, which corresponds to the choked flow regime. A moderate cavitation number (between inception and supercavitation flow regimes), where the gas phase is elongated along the nozzle are, leads to the developed flow regime (Fig. 2).

**Fig. 3:**
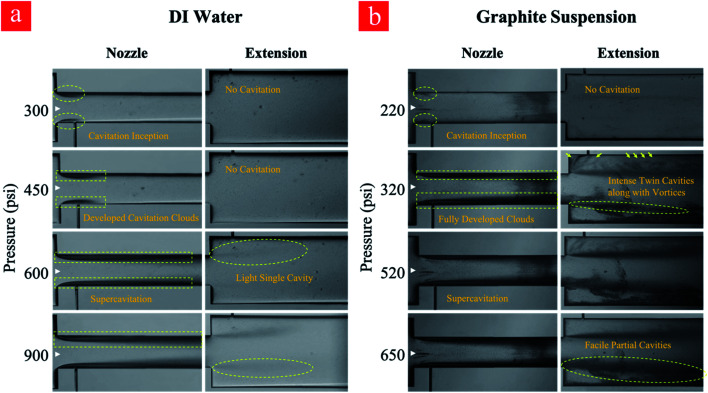
Different cavitation flow patterns at various upstream pressures for (a) water and (b) graphite suspension.

As can be seen in Fig. 3, the upstream pressure for the case of graphite suspension is lower for the identical cavitating flow patterns. This observation can be explained by the increased number of heterogeneous nucleation sites in this case of suspensions. The presence of graphite particles in the working fluid acts as a solid/liquid interface. The micro-scale roughness elements on the surface of the graphite particles act as further heterogeneous nucleation sites, which can facilitate the inception and development of the cavitating flows.

**Fig. 4:**
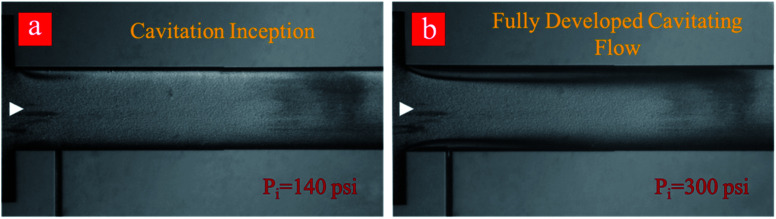
Inception and developed cavitating flow pattern for the graphene suspension after 80-cycles of hydrodynamic cavitation. (a) The inception begins at 140 psi, and (b) the fully developed cavitation flow pattern at 300 psi.

Regarding the application of the fabricated microfluidic device in this study, developed flow regime corresponding to the upstream pressure of 650 psi is suitable for cyclic treatment of the graphite suspension and subsequent exfoliation due to the optimum conditions regarding the input power and output of the process. The experiments include 0–80 cycles so that the graphite suspension is thus treated with cavitating flows. Cavitating flows corresponding to inception and developed flow after the 80^th^ cycle are shown in Fig. 4. As shown in this figure, the cavitation inception decreases from 220 psi (first cycle) to 140 psi at the 80^th^ cycle, while fully developed cavitating flow is seen at the upstream pressure of 300 psi. This indicates that more heterogeneous sites as a result of the fine exfoliation are formed inside the introduced suspension, and the nucleation is triggered more vigorously after the 80^th^ cycle.

### 3.2. Characterization of graphene nanosheets

In the hydrodynamic cavitation-assisted graphene production process, the starting graphite dispersion was circulated through the system to evaluate the effect of the number of cycles on graphene production. The pre-defined cycles of 20, 40, 60, and 80 were used to study this effect. For example, to prepare a graphene-containing solution *via* 20-cycles, the starting graphite dispersion was circulated 20 times through the hydrodynamic cavitation system, and the obtained solution of the graphene nanosheets was analyzed using spectroscopic and microscopic techniques. To maintain the homogeneity in the produced graphene nanosheets, a sequential centrifugation method was developed and applied for all samples. In this method, the graphene dispersions after the hydrodynamic cavitation treatment were first centrifuged at 2000 rpm for 1 hour; thus, the exfoliated graphene nanosheets and small fragments of graphite were obtained in the supernatant solution (S2U). This supernatant was subjected to a second centrifugation process at 3000 rpm for 1 hour to remove large particles and to isolate the highly exfoliated stable graphene nanosheets (S3U). Fig. 1b depicts a schematic for the isolation of the stable graphene nanosheets.

The isolated graphitic materials and the starting graphite dispersion were first characterized by Raman spectroscopy to evaluate the effect of the hydrodynamic cavitation on the exfoliation of graphite flakes. Raman spectroscopy is a versatile tool to analyze the structure of carbon nanomaterials, including carbon nanotubes^49,50^ and graphene.^51,52^ In a typical Raman spectrum of graphene, there are three commonly reported peaks as D, G, and 2D bands at around 1350, 1580, and 2700 cm^−1^, respectively.^53^ The D band in the spectrum is related to the structural disorders, edges, and topological defects in the flakes. The area ratio of D-band to G-band (*A*_D_/*A*_G_) is often used to define the relative amount of surface defects on the graphene.^54,55^ Besides, the 2D-band for graphene is attributed to two-phonon double resonance and can be used as a measure to evaluate the number of layers in the graphene nanosheets. More specifically, the intensity ratio of 2D-band to G-band band (*I*_2D_/*I*_G_) is an indication for the number of layers of graphene.

**Fig. 5:**
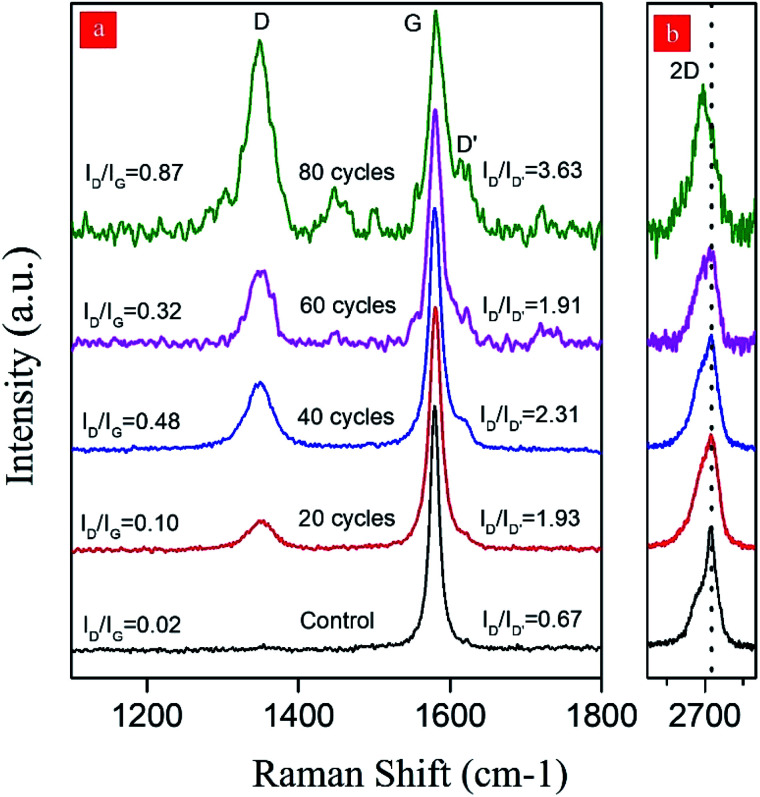
The normalized and offset Raman spectra (at G-band) of the produced graphene nanosheets after different cavitation cycles within the reactor. (a) D-, G- and D′-band region of the Raman spectra (b) 2D-band region. The dotted line in (b) shows the shift in the position of the 2D-band after 80-cycles of cavitation.

Fig. 5 displays the Raman spectra of S3U-20, S3U-40, S3U-60, S3U-80, and the starting graphite dispersion. It is known that the position and shape of the 2D-band are highly sensitive to the number of graphene layers (less than 10 layers) because of the relations of peak activation parameters of Raman mode and band structure.^56^ No significant change is observed in the maxima of the 2D-band of the S3U-20, S3U-40, and S3U-60 compared to the starting graphite dispersion (2D-band ∼2716 cm^−1^). However, there is a significant downshift (∼25 cm^−1^) in the maximum of the 2D-band for the S3U-80 and, the band appeared at 2692 cm^−1^. In agreement with the literature,^52^ the observed shift can be attributed to the formation of bilayer graphene nanosheets after 80-cycles of hydrodynamic cavitation. Furthermore, the enhanced *I*_2D_/*I*_G_ ratio after 80-cycles further supports the formation of a few layer graphene nanosheets.^57^

When the defect density was analyzed, almost no defect was observed for the starting graphite dispersion. In contrast, the isolated graphene nanosheets have *I*_D_/*I*_G_ ratios of 0.10, 0.48, 0.32, and 0.87 for the S3U-20, S3U-40, S3U-60, and S3U-80, respectively, suggesting a gradual defect formation.^58^

The nature of defects in graphene was previously studied,^59^ and it was shown that the intensity ratio between the D-band and D′-peak (at *ca.* 1620 cm^−1^) could be used as a measure to probe the nature of the defects. In general, this ratio (*I*_D_/*I*_D′_) was found to be ∼13 for sp^3^-defects, while it was ∼7 and ∼3.5 for vacancy-like defects and boundaries in graphite, respectively. After the application of hydrodynamic cavitation, the intensity of D′-peak gradually increases with an increase in the number of cycles. In parallel, as above-mentioned, the intensity of D-band ∼1620 cm^−1^ also gradually increases. The isolated graphene nanosheets have *I*_D_/*I*_D′_ of 1.93, 2.31, 1.91, and 3.63 for the S3U-20, S3U-40, S3U-60, and S3U-80, respectively. From the observed ratios, it can be concluded that the hydrodynamic cavitation creates surface defects on the exfoliated graphene nanosheets, and the defect density becomes more pronounced after 80-cycles of cavitation. However, it is worth pointing out that the calculated *I*_D_/*I*_D′_ ratios are lower than the ratios reported for the graphene nanosheets with sp^3^ and vacancy-like defects.

**Fig. 6:**
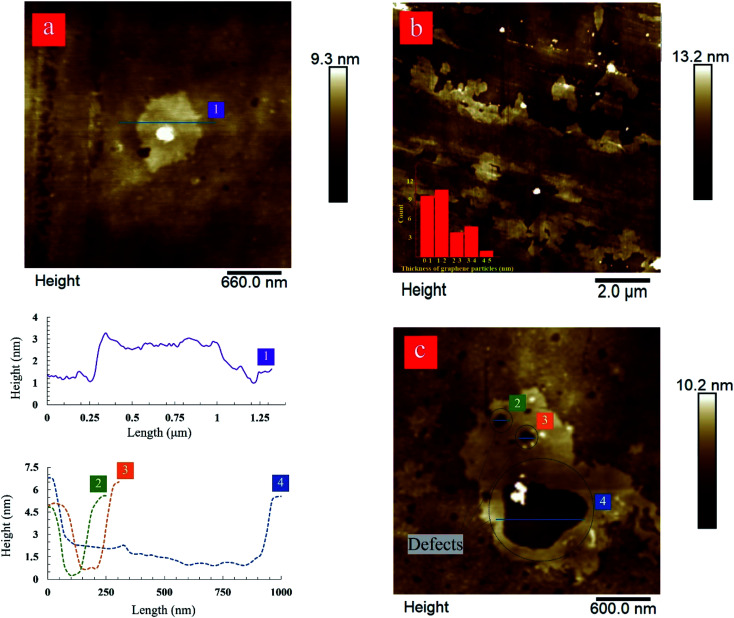
AFM images of monolayer and few layers of graphene sheets. (a) A graphene sheet with a thickness about ∼1 nm after 80-cycles, (b) the exfoliated graphene nanosheets large in lateral size after 60-cycles of treatment, and (c) a graphene sheet with defects, which were formed by exposure to excessive bubble collapse after 60-cycles.

The atomic force microscopy (AFM) characterization was used to determine the size and thickness of produced graphene nanosheets. The results complement the Raman spectroscopy data. AFM image and height profile of the produced graphene nanosheets after 80-cycles of hydrodynamic cavitation further confirm the exfoliation of graphite flakes into bi-layer graphene nanosheets having a thickness value of ∼1 nm (Fig. 6a). The thickness range of the produced graphene nanosheets after 60-cycles is approximately between 1.2 and 2.5 nm, which is considered as ≤3 layer graphene (Fig. 6b).^60^ The lateral size of the analyzed nanosheets is in the range of 1–5 μm. Furthermore, close inspections on the AFM image of the few-layer graphene nanosheets produced after 60-cycles display large holes, which vary in sizes between 100 to 600 nm (Fig. 6c). The presence of these holes correlates well with the *I*_D_/*I*_G_ ratios obtained by Raman spectroscopy, suggesting the formation of defects at the edges. These defects are not surprising since the exfoliated graphene nanosheets were subjected to intense cavitation energy. As in the LPE process, the formation of defects in the forms of edges and topological defects is unavoidable because these types of defects need lower formation energy. Furthermore, the size of the defects is believed to be related to the size of bubbles, which varies from hundreds of nanometers to micrometers.

UV-Vis spectroscopy was performed to assess the concentration of the isolated graphene nanosheets and the starting graphite dispersion (see ESI Fig. S1†). In agreement with the literature, the absorption spectra of the isolated graphene nanosheets (S3U-20 to 80) are featureless in the measurement range. Compared to the concentration of the starting graphite dispersion (25 μg mL^−1^), the concentrations of the obtained graphene nanosheets are calculated as ∼2.1 (S3U-20), 1.1 (S3U-40), 1.0 (S3U-60), and 1.1 (S3U-80) μg mL^−1^ using the molar absorption coefficient of 3620 mL mg^−1^ m^−1^ at the wavelength of 660 nm for the graphene in water.^61^ Using the concentrations of the isolated graphene nanosheets, the process efficiencies after 20-, 40-, 60-, and 80-cycles are calculated as 8.4%, 4.8%, 4%, and 4.4%, respectively. It is worth noting that the efficiency of the hydrodynamic cavitation-assisted graphene production process after 40-cycles is higher than the previously reported study.^58^ However, the yield of exfoliated graphene showed a fast decrease after 20-cycles. The observed concentration loss may be related to the trapping of exfoliated graphene in the cavities and the porosities of the system. The efficiency of the system can be improved by reducing the length of the pipes, porosities of the exposed surfaces, and using a closed-loop system. Moreover, the UV-Vis analysis of a control sample prepared by centrifuging the starting graphite dispersion exhibits almost no absorbance at 660 nm (1.4 μg mL^−1^). This result indicates that the stability of the isolated graphene nanosheets is higher than that of the starting graphite dispersion, probably due to the size shortening of graphite flakes.

**Fig. 7:**
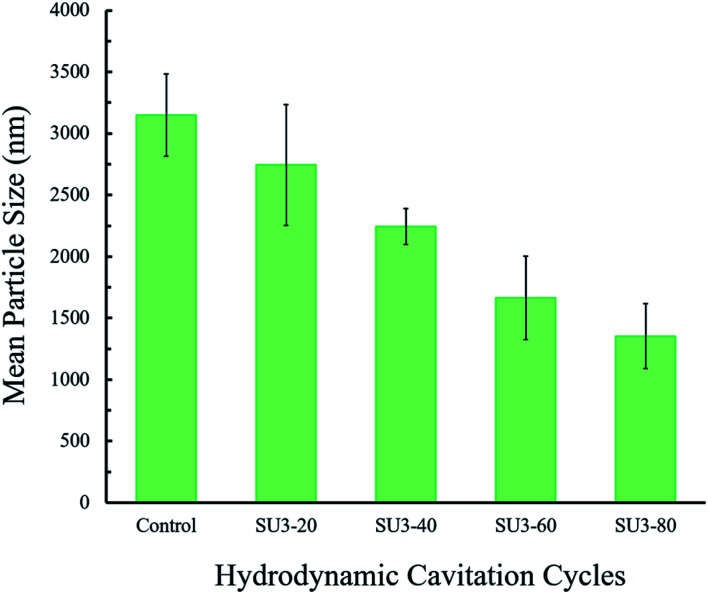
The measured average particle size of the hydrodynamic cavitation (20, 40, 60, and 80-cycles) treated and non-treated graphene nanosheets after two centrifugation steps.

The size distribution of the isolated graphene nanosheets was studied using the dynamic light scattering (DLS) technique. The DLS results of the centrifuged samples of carbon-based (graphite/graphene) colloids at the end of different hydrodynamic cavitation cycles are demonstrated in Fig. 7. From the DLS data, it can be deduced that the mean diameter size of the particles gradually decreases with the increase in the number of hydrodynamic cavitation cycles. The mean particle size of the flakes in the starting graphite dispersion after two sets of centrifugation steps is measured as 3150 (±335) nm. Nevertheless, the measured particle sizes of S3U-20, S3U-40, S3U-60, and S3U-80 are 2744, 2242, 1664, and 1353 nm, respectively. The differences between the mean particle sizes are well-correlated with the number of hydrodynamic cavitation cycles. Complementary optical microscopy images are illustrated this fragmentation and size differences (see ESI Fig. S2†).

**Fig. 8:**
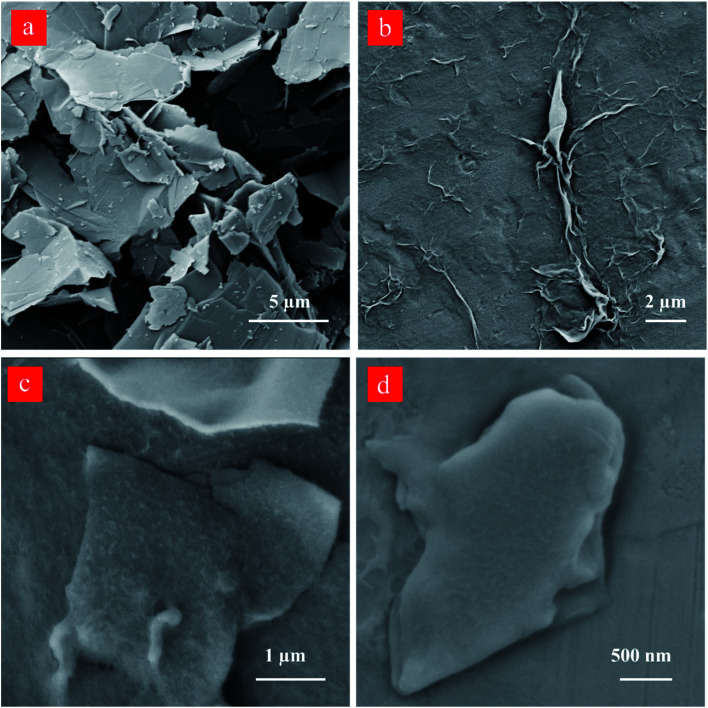
SEM images of (a) the starting graphite dispersion, the graphene nanosheets obtained after sequential centrifugation of (b) 60-cycles in low magnification, (c) 60-cycles in higher magnification, and (d) 80-cycles cavitation-treated graphite dispersion.

The scanning electron microscopy (SEM) image of the starting graphite dispersion displays large flakes having a lateral dimension of over ∼5 μm (Fig. 8a). The lateral flake sizes of the isolated graphene sheets after 60 and 80-cycles decrease to ∼4 and ∼3 μm, respectively. The treatment by the reactor under the developed cavitating flow pattern causes changes in the graphite lateral size, and the SEM results are in good agreement with the DLS size distribution as well as the AFM data.

**Fig. 9:**
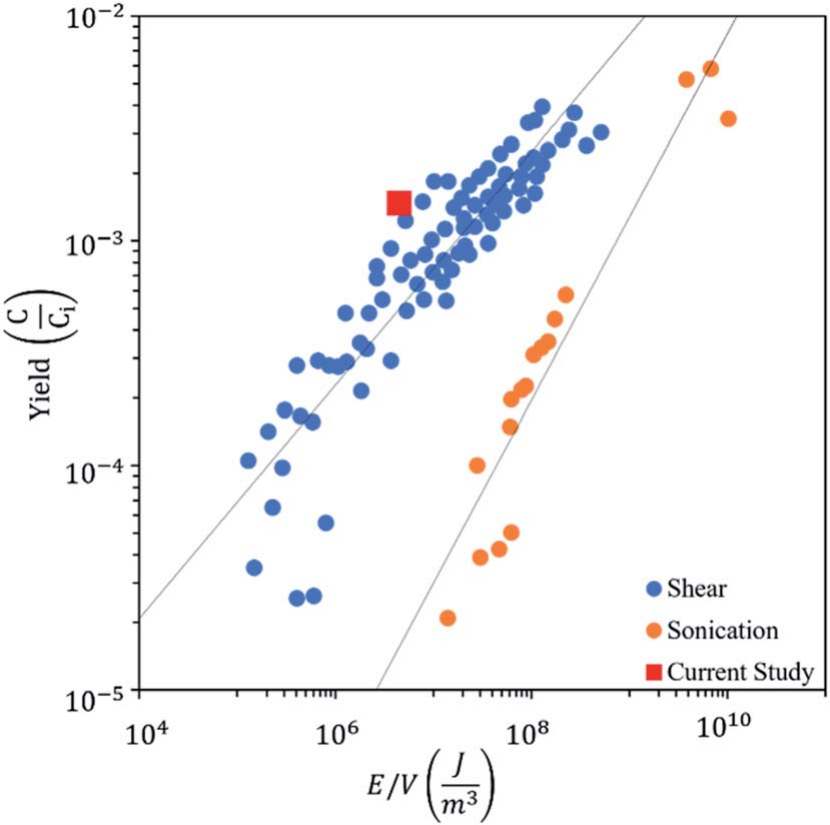
Graphene exfoliation yield as a function of energy density with the use of shear, sonication, and hydrodynamic cavitation exfoliation.

The residence time of the fluid in the hydrodynamic cavitation device is rather short (2.9 × 10^−6^ s). Therefore, the energy density (*E* = J m^−3^) can be calculated using the pressure differential along the channel.^62^ All of the cycles were performed at 650 psi (4.48 × 10^6^ J m^−3^) for having consistent results. However, the pressure sufficient to have graphene exfoliation decreases to 300 psi (2.06 × 10^6^ J m^−3^) with the number of cycles due to exfoliation and fragmentation of graphite powder in lower cycles, which provides active sites for nucleation of the cavitating bubbles. Fig. 9 shows the graphene exfoliation yield as a function of energy density and compares hydrodynamic cavitation with the sonication and shear methods reported in the literature.^63,64^ The results on hydrodynamic cavitation show a higher yield by consuming a lower energy density for graphene exfoliation. As a well-known technique for graphene exfoliation, sonication consumes 25–540 watts of power (5 times more than hydrodynamic cavitation) for an extensive amount of time (3000 times more than hydrodynamic cavitation) to exfoliate a fraction of 1 liter of graphite solution.^61,65–68^ Although the size of used graphite powders limits the hydrodynamic cavitation method, 300 times more energy is required to achieve the same yield for the sonication method.

## 4. Conclusions

In this study, first, the effects of the thermophysical properties of the working fluid (presence of graphite flakes) on cavitating flows were visualized and studied. Then, the impact of cavitating flows on graphite exfoliation was investigated. This method does not involve any surfactants or dispersion agents. According to the results, the suspensions with graphite flakes led to an increase in the number of the sites of heterogeneous bubble nucleation and to a decrease in the upstream pressure needed for cavitation inception and a developed cavitating flow pattern. Fragmentation of flakes and then exfoliation of layers was observed after exposures of 60–80 hydrodynamic cavitation cycles inside a microfluidic device and were rigorously characterized with different methods. With the implementation inside the reactor, it is possible to have a green, scalable, cost-effective, and energy-efficient process. The produced graphene nanosheets (lateral size ≥500 nm; thickness ∼1.2–2.5 nm) meet the requirements well in many applications such as bioengineering, composites, and electronic devices. The results on hydrodynamic cavitation show a higher yield compared to the sonication and shear methods for graphene exfoliation.

## Data availability

The data that support the findings of this study are available from the corresponding author upon reasonable request.

## Conflicts of interest

The authors declare that they have no conflict of interest.

## Supplementary Material

RA-011-D1RA03352B-s001

RA-011-D1RA03352B-s002
