# Supplementary material for: An ecologically friendly process for graphene exfoliation based on the “hydrodynamic cavitation on a chip” concept
Source: RSC Adv. 2021 May 18;11(29):17965–75. doi: 10.1039/d1ra03352b (PMC9033250; doi:10.1039/d1ra03352b)
Supplement: RA-011-D1RA03352B-s002 [file RA-011-D1RA03352B-s002.pdf]

Electronic Supplementary Material (ESI) for RSC Advances.
This journal is © The Royal Society of Chemistry 2021

Supplementary information for:

An Eco-friendly Microreactor Based on the "Hydrodynamic Cavitation on a Chip" Concept for Graphene Exfoliation

Mohammad Jafarpour[1,2], Araz Sheibani Aghdam[1,2], Moein Talebian Gevari[3], Ali Koşar[1,2,4], Mustafa Kemal Bayazıt[1,2], Morteza Ghorbani[1, 4, 5*]

[1]Sabanci University Nanotechnology Research and Application Center, 34956 Tuzla, Istanbul, Turkey

[2]Faculty of Engineering and Natural Science, Sabanci University, 34956 Tuzla, Istanbul, Turkey

[3]Division of Solid State Electronics, Department of Electrical Engineering, The Ångström Laboratory, Uppsala University, 75237 Uppsala, Sweden

[4]Center of Excellence for Functional Surfaces and Interfaces for Nano-Diagnostics (EFSUN), Sabanci University, Orhanli, 34956, Tuzla, Istanbul, Turkey

[5]Department of Biomedical Engineering and Health Systems, KTH Royal Institute of Technology, SE-141 57 Stockholm, Sweden

[*]Corresponding Author: Morteza Ghorbani (mortezag@kth.se)

This supplementary information contains SD1-SD2.

Supplementary Data 1:

The first supplementary date is about the monitored files of flow patterns of the suspension at 140 psi and 300 psi. These avi files are added separately. S1-1 shows the cavitation inception point at 140 psi and S1-2 shows the fully developed flow pattern at the 300 psi.

Supplementary Data 2:

The concentration of suspensions was calculated using Beer-Lambert's equation.

$$A= \varepsilon.L.C$$

where A is absorbance, ε is the molar absorption coefficient, L is path length of the measuring beam in the sample and c is the concentration. The molar absorption coefficient of graphene in water was considered as 3620 $ml.mg^{-1}.m^{-1}$ at the wavelength of 660 nm for the graphene suspension [1]. Figure S1 shows the UV-Vis spectra of the samples which treated different times through the microreactor. Since suspensions stability is not a concern in this study, and any kind of surfactants or stabilization agents were not used in the preparation of the suspensions, therefore, sedimentation of the particles in liquid medium are very likely. This is causing some problems in calculation, but as particles get smaller, their stability also improves. The yield of the process was calculated for samples (S3U-20, S3U-40, S3U-60, and S3U-80). This data comes out by considering the initial concentration as 25 µg mL^{-1} and take the treated concentration of samples from UV-Vis spectra.

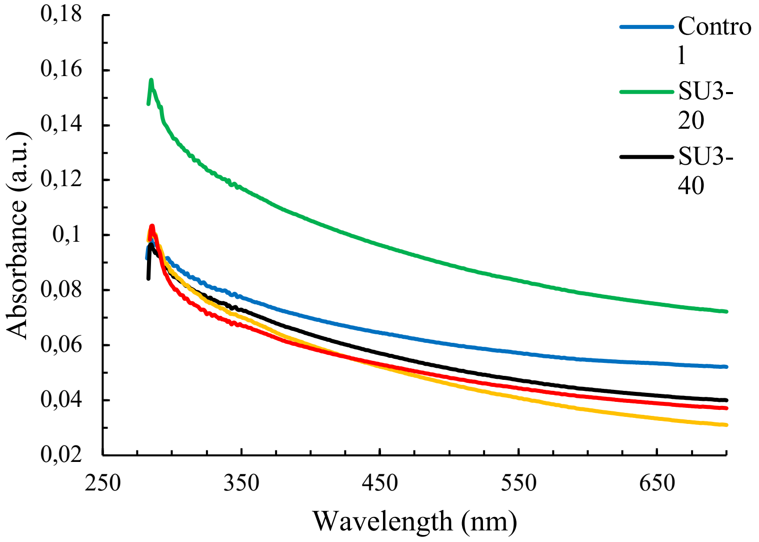

Figure S1: UV-Vis spectra of the control sample and the sample which pass through the microreactor 20, 40, 60, and 80 times.

Reference:

[1] U. Khan, A. O'Neill, M. Lotya, S. De, J.N. Coleman, High-concentration solvent exfoliation of graphene, Small. 6 (2010) 864–871. https://doi.org/10.1002/smll.200902066.
